# Safety of Abatacept in Italian Patients with Rheumatoid Arthritis and Interstitial Lung Disease: A Multicenter Retrospective Study

**DOI:** 10.3390/jcm9010277

**Published:** 2020-01-19

**Authors:** Giulia Cassone, Andreina Manfredi, Fabiola Atzeni, Vincenzo Venerito, Caterina Vacchi, Valentina Picerno, Federica Furini, Gian Luca Erre, Paola Tomietto, Anna Laura Fedele, Giovanni Della Casa, Valeria Nucera, Chiara Giannitti, Carlo Salvarani, Marco Sebastiani

**Affiliations:** 1 Clinical and Experimental Medicine PhD Program, University of Modena and Reggio Emilia, 41121 Modena, Italy; giulia.cassone@unimore.it (G.C.); caterina.vacchi@unimore.it (C.V.); 2 Chair and Rheumatology Unit, University of Modena and Reggio Emilia, Azienda Ospedaliero-Universitaria Policlinico di Modena, 41121 Modena, Italycarlo.salvarani@unimore.it (C.S.); 3 Rheumatology Unit, University of Messina, 98121 Messina, Italy; atzenifabiola@hotmail.com (F.A.);; 4 Rheumatology Unit, Interdisciplinary Department of Medicine, University of Bari, 70121 Bari, Italy; vincenzo.venerito@gmail.com; 5 Rheumatology Institute of Lucania (IReL) and Rheumatology Department of Lucania, San Carlo Hospital of Potenza and Madonna delle Grazie Hospital of Matera, 85100 Potenza, Italy; valepicerno@tiscali.it; 6 Department of Medical Sciences, Division of Rheumatology, Santa Anna University Hospital, 44100 Ferrara, Italy; fede.furini@gmail.com; 7 Rheumatology Unit, Azienda Ospedaliero-Universitaria di Sassari, 07010 Sassari, Italy; gianluca.erre@aousassari.it; 8 Department of Clinical Medicine, Rheumatology Unit, Azienda Sanitaria Universitaria Integrata di Trieste, 34121 Trieste, Italy; paola.tomietto@asuits.sanita.fvg.it; 9 Rheumatology Unit, Policlinico Gemelli Foundation, Catholic University of the Sacred Heart, 00168 Rome, Italy; 10 Radiology Unit, University of Modena and Reggio Emilia, Azienda Ospedaliero-Universitaria Policlinico di Modena, 41121 Modena, Italy; 11 Rheumatology Unit, University of Siena, 53011 Siena, Italy

**Keywords:** rheumatoid arthritis, interstitial lung disease, safety, therapy, abatacept

## Abstract

Background: Treatment of rheumatoid arthritis (RA)-related interstitial lung disease (ILD) is challenging, and many conventional and biologic disease-modifying anti-rheumatic drugs (DMARDs) have been associated with ILD development or progression. The aim of this multicentric retrospective study was to analyze the evolution of ILD in Italian RA-ILD patients treated with abatacept (ABA). Methods: All RA-ILD patients treated with ABA for at least six months were retrospectively evaluated. Serology, previous and concurrent therapies, chest high-resolution computer tomography (HRCT), forced vital capacity (FVC), and lung diffusion of carbon monoxide (CO, DLCO) were collected. Results: Forty-four patients were included; HRCT, FVC, and DLCO were analyzed at baseline, at one year, and at the end of follow-up. A remission or a low disease activity of RA was reached in 41/44 patients. Overall, FVC and DLCO remained stable or increased in 86.1% and 91.7% of patients, respectively, while HRCT was stable or improved in 81.4% of them. Previous and concurrent treatments, in particular, methotrexate, serology, age, sex, joint and lung disease duration were not associated with the outcome at univariate analysis. Conclusion: The management of RA-ILD patients remains a critical unmet medical need. Waiting for prospective controlled studies, ABA has shown a good safety profile in our cohort of Italian RA-ILD patients.

## 1. Introduction

Interstitial lung disease (ILD) is a severe extra-articular manifestation of rheumatoid arthritis (RA), deeply impacting patients’ quality of life, overall prognosis, and survival [1,2].

Although the prevalence of ILD in RA patients is unclear, this disease is responsible for decreased quality of life and progressive chronic disability in a significant proportion of patients and10%–20% of deaths associated with the disease, with an estimated mean survival of 5–8 years [3,4,5,6].

The pathogenesis of RA-associated ILD (RA-ILD) remains unclear [7], but both genetic and environmental factors have been investigated.

Moreover, almost all the conventional and biologic disease-modifying anti-rheumatic drugs (DMARDs) have been associated with ILD development or progression [8,9,10].

Waiting for controlled studies, the therapeutic strategy for RA-ILD patients is still debated and empirical [2,7,10,11,12]; in fact, the treatment of joint involvement in this subgroup of patients should be effective without worsening the lung manifestation of the disease.

Abatacept (ABA) is a soluble fusion protein comprising cytotoxic T-lymphocyte-associated protein 4 and an Fc portion of immunoglobulin G1, which inhibits T-lymphocyte co-stimulation and is approved for the treatment of moderate to severe RA [13,14]. In the last years, an increasing interest in ABA for use in RA-ILD treatment has emerged, based on its capacity to improve ILD in mice models [15,16]. Recently, some authors described the possible effectiveness and safety of ABA in the treatment of patients with RA-ILD [17,18,19,20,21,22].

In this multicenter retrospective study, we analyzed the evolution of ILD in a population of RA patients treated with ABA. 

## 2. Patients and Methods

RA-ILD patients treated with ABA attending the rheumatology units of 8 Italian centers were enrolled in this national multicentric study. All RA patients diagnosed between 2012 and 2018 according to the 1987 or 2010 classification criteria, who underwent ABA therapy for at least 6 months, were retrospectively evaluated to identify patients with ILD [23,24].

The study was approved by the local Institutional Review Board “Comitato Etico Area Vasta Nord” (approval number: 109/2019/OSS/AOUMO).

The diagnosis of ILD was made by mean of high-resolution computer tomography (HRCT) of the chest, and the different ILD patterns were classified according to the criteria of the American Thoracic Society/European Respiratory Society International Multidisciplinary Consensus Classification of the Idiopathic Interstitial Pneumonias [25] as follows: definite or probable usual interstitial pneumonia (UIP), non-specific interstitial pneumonia (NSIP), organizing pneumonia (OP), and mixed patterns. 

HRCT images were scored according to previous classifications. The areas of reticulation and honeycombing, ground glass, and consolidation were calculated at 6 anatomical levels: the arch of the aorta; the carina; the pulmonary venous confluence; a point halfway (i) between the pulmonary venous confluence and 1 cm above the dome of the right hemidiaphragm; (ii) 1 cm above the dome of the right hemidiaphragm; (iii) 2 cm below the dome of the right hemidiaphragm [26].

Rheumatoid factor (RF) was determined by nephelometry; a standard commercial enzyme-linked immunosorbent assays (ELISA) was used to detect anti-cyclic citrullinated peptides antibodies (ACPA). 

The results of pulmonary function tests (PFT) were expressed as percentages of the predicted value of each parameter and corrected for age, gender, and height. Pulmonary function was considered as normal if forced vital capacity (FVC) was ≥80% of the predicted values. Single-breath diffusing capacity of the lung for carbon monoxide (DLCO-SB) was used to assess gas transfer. The last HRCT and the last PFT performed before starting ABA were recorded as baseline.

### 2.1. Outcome Variables

A variation of 10% of FVC and 15% of DLCO compared to baseline values was considered clinically significant [27]. Improvement, worsening, or stability of HRCT was centrally re-evaluated in a blinded manner by an experienced thoracic radiologist (GDC). PFTs were collected at baseline and periodically assessed, and the last available value for all patients was recorded (within three months from the end of follow-up).

### 2.2. Statistical Analysis 

Results were expressed as median and interquartile range (IQR). Unpaired or paired nonparametric tests (Mann–Whitney or Wilcoxon test, respectively) were used to compare continuous variables. A *p* value less than 0.05 was considered significant. Statistical analyses were performed using the SPSS statistical software, version 17.0 (SPSS Inc., Chicago, IL, USA) [28].

## 3. Results

We enrolled 44 RA-ILD patients (32 females and 12 males, median age 65 years, IQR 11) treated with ABA. The drug was administered at the standard dose, both intravenously (every four weeks) and subcutaneously (125 mg weekly). At the time of ABA initiation, the median of RA duration was 89 months (IQR 142), while ILD predated by a median of 20 months (IQR 58). Eight (18.2%) patients were smokers at baseline, while another 10 had quit smoking in the previous 10 years. A chronic obstructive pulmonary disease was recorded in 10 patients (22.7%).

All patients underwent HRCT in the previous 12 months before the beginning as well as at the end of the ABA therapy, while PFTs were available in 39/44 patients (88.6%).

The baseline characteristics of our patients are summarized in Table 1.

The median follow-up was 26.5 months (range 6–116, IQR 38). A high percentage of patients were positive for RF (38/44 patients, 86.4%) and for ACPA (40/44, 90.1%). 

In all but three RA patients, we assessed a remission or a low disease activity at the end of follow-up.

### 3.1. Previous Treatments

Before the assumption of ABA, all patients experienced therapies with other synthetic and/or biologic DMARDs.

In particular, all patients but five were previously treated with methotrexate (MTX) or leflunomide (LFN), namely, 32 (72.7%) with MTX and 20 (45.5%) with LFN. Twelve patients were previously treated with both drugs, alone or in combination. 

ABA was the first biologic DMARD in 19 patients (43.2%), and the second in another 15 (34%). Twenty-five subjects were previously treated with other biologic DMARDs, in particular 19 subjects (43.2%) with a tumor necrosis factor inhibitor (TNFi), 9 (20.5%) with tocilizumab, 5 (11.4%) with rituximab, and 3 (6.8%) with a Janus kinases inhibitor.

### 3.2. Current Treatments

Only four patients (9.1%) were treated with intravenous ABA, while three (6.8%) were switched from intravenous to subcutaneous administration. ABA was prescribed in combination with MTX to 17 patients (38.6%) and with other DMARDs to 16 patients (36.4%); monotherapy with ABA was administered to the other 11 patients (25%) in combination with a low dose of steroids. Finally, a low dose of prednisone (usually ≤5 mg daily) was prescribed to 33 patients (75%).

### 3.3. ILD Radiologic Patterns

All patients had an HRCT in the 12 months before starting ABA. UIP and NSIP were the two prevalent HRCT patterns (43.2% and 50% for UIP and NSIP, respectively), while combined pulmonary fibrosis and emphysema (CPFE) were described in two patients (4.5%) and organizing pneumonia in one (2.3%).

### 3.4. Pulmonary Function Tests

PFTs were available at baseline in 39/44 patients. The median of FVC was 89% (IQR 18); FVC was normal at baseline in 82.1% of patients (32/39). DLCO was available in 38/44 patients and was normal in less than 50% of patients (44.7%, 17/38), with a median of 66.4% (IQR 34.5).

### 3.5. Evolution of Lung Function and HRCT

**Figure 1:**
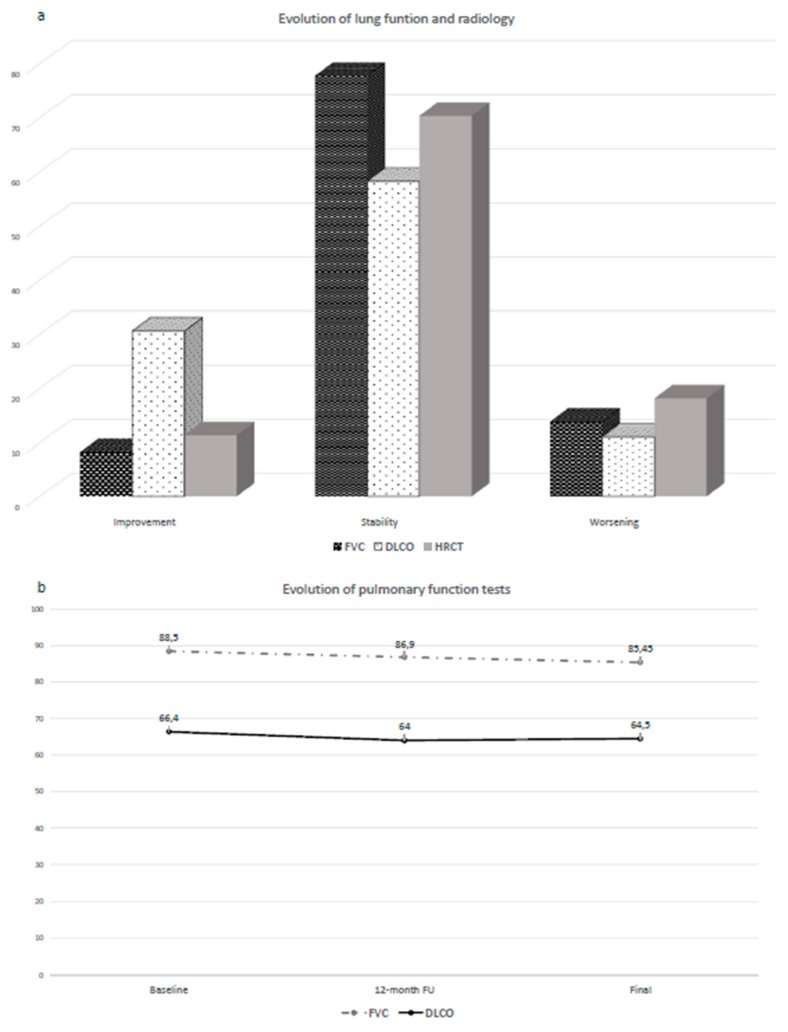
Evolution of lung function and radiology during follow-up. (**a**) During follow-up, FVC remained stable in 77.8% of patients, improved in 8.3% and worsened in 13.9%. During follow-up, DLCO remained stable in 58.3% of patients, worsened in 11.1%, and improved in 30.5%. HRCT was stable in 70.4% of cases, worsened in 18.2%, and improved in 11.4%. (**b**) Evolution of lung function. The median of FVC was 88.5%, 86.9% and 85.45% at baseline, after one year and at the end of follow-up, respectively. The median of DLCO was 66.4%, 64% and 64.5% at baseline, after one year and at the end of follow-up, respectively. FVC: forced vital capacity; DLCO: diffusing capacity of the lung for carbon monoxide; HRCT: high resolution computed tomography.

The changes of respiratory function and radiology are described in Figure 1a,b.

PFTs were available after one year and at the end of the follow-up in 36 patients. The median of FVC was 86.9% (IQR 28.8) and 85.45% (IQR 21.5) after one year and at the end of follow-up, respectively. After one-year follow-up, FVC remained stable in 28/36 patients (77.8%), improved in three (8.3%) and worsened in five (13.9%). The trend of FVC did not change at the end of follow-up: FVC remained stable, improved, and worsened in 28, 3, and 5 patients, respectively.

The median of DLCO was 64% (24.3) and 64.5% (27.5) after one year and at the end of follow-up, respectively. After one year, DLCO remained stable in 50% of patients (18/36), worsened in 11.1% (4/36), and improved in 38.9% (14/36). At the end of follow-up, three patients initially improved, then showed a decrease of DLCO up to the baseline values.

HRCT was performed at the end of the follow-up in all patients and was stable in 31/44 cases (70.4%), worsened in 8 (18.2%), and improved in 5 patients (11.4%). Worsening was recorded in six patients with UIP and two with NSIP, while at the same time, improvement was observed in four patients with NSIP and only one with UIP.

On the whole, HRCT in patients with UIP worsened in 6 cases, remained stable in 12, and improved in one; NSIP remained stable in 16 patients, improved in four, and worsened in two.

Univariate analysis did not show any differences in patients with worsening, stability, or improvement of lung disease, in relation to smoking habit, previous therapy with MTX or LFN, previous biologic DMARDs, combination therapy with MTX, positivity for ACPA or RF, age at disease onset, and joint and lung disease duration (Table 2).

A trend for a worst FVC evolution was observed in male patients (*p* = 0.07).

During the follow-up period, ABA was withdrawn in only four patients, because of loss of efficacy on RA in three patients and a non-respiratory infectious adverse event in the fourth case.

## 4. Discussion

Treatment of RA-ILD remains challenging, given the lack of randomized controlled clinical trials and the possible role of DMARDs in lung toxicity and in the development of acute exacerbation (AE) of ILD [2,8,9,10,11,12]. 

Recently, some authors noted in case reports and retrospective studies the possible effectiveness and safety of ABA for the treatment of RA patients complicated by ILD, reporting stability of lung function during the follow-up period and the absence of severe adverse events [17,18,19,20,21,22]. In particular, no cases of AE of ILD were described during the therapy (only one case of AE two months after ABA discontinuation has been described in the literature [29]).

ABA was associated with the improvement of FVC and DLCO in 20% and 25%, respectively, of 63 RA-ILD patients from a Spanish retrospective cohort [21], while Mochizuki described an improvement in 14.5% of 55 RA-ILD patients. Worsening of ILD was associated with the concomitant use of MTX at multivariate logistic regression analysis [22].

Moreover, Nakashita recorded new appearance in 3% and worsening of pre-existing ILD in 24% of RA patients treated with TNFi, compared with no events in the ABA and tocilizumab group [19,30]. On the other side, Curtis et al. found no significant differences in the risk of ILD incidence and its related complications in a large cohort of patients receiving second-line biologic therapy after a first-line treatment with TNFi among patients exposed to tocilizumab, rituximab, or ABA compared with TNFi therapies [20].

Kurata et al. recently found that ABA was an independent protective factor for RA-ILD exacerbation after the initiation of bDMARDs [31].

Finally, a small clinical trial to assess the safety of ABA in patients with RA-ILD is ongoing (APRIL study, NCT03084419). The investigators emphasize that this is a small clinical trial to assess the feasibility of performing a larger randomized controlled trial [32].

Our data confirm the safety of ABA in the treatment of RA patients, also for patients with lung involvement. MTX seemed to be safe in our RA-ILD patients, though, due to the low number of patients, the possible role of MTX as a factor associated with ILD evolution could not be fully evaluated; prospective studies should be scheduled in this regard. 

In a Japanese series, MTX has been suspected to be involved in lung disease progression [22]. In contrast, we systematically evaluated previous and concurrent therapy with ABA without highlighting any role for MTX or other conventional DMARDs in the progression of ILD in Italian RA patients. Moreover, the role of MTX in lung toxicity has been recently analyzed in some meta-analyses and registry-based studies, suggesting a safe profile of MTX in RA-ILD patients. In particular, in a recent meta-analysis MTX was not associated with infectious and non-infectious pulmonary adverse events, including the development of ILD. Moreover, of interest, no MTX-related pneumonitis has been reported in controlled studies after 2001 [33]. 

Lastly, the analysis of two large multicenter RA inception cohorts—the early rheumatoid arthritis study (ERAS) and the early rheumatoid arthritis network (ERAN)—recruiting from 1986 to 2012 with review up to 25 years, concluded that MTX treatment was not associated with an increased risk of RA-ILD diagnosis [34]. 

Despite the positive data, the exact role of MTX is still questioned and i can be defined only in prospective long-term studies.

The main limit of our study is represented by its retrospective design, but the rigorous recording of PFT and the centrally re-evaluation of HRCT ensure the quality of our data.

The available data did not allow us to postulate as to the efficacy of ABA in the treatment of ILD in RA patients, but our data further reinforce our knowledge about the safety of ABA in the management of RA complicated by ILD. Moreover, ILD is associated with a high risk of infectious complications, and the safety profile of ABA with regard to infection could represent another element encouraging the use of this drug in RA-ILD patients [35]. Other biologic DMARDs, such as interleukin 6 and Janus kinases inhibitors, seem to be associated with a good safety profile in RA patients complicated by ILD [36,37,38,39].

In conclusion, the management of RA-ILD patients remains a critical unmet medical need. As we await prospective controlled studies of patients with RA-ILD, biologic DMARDs such as ABA that have demonstrated a good safety profile in this specific population [17,18,19,20,21,22] should be the preferred treatment.

Finally, an early diagnosis of ILD in RA patients should be encouraged to increase our knowledge of the natural history of ILD, its possible predictive factors, and the possible involvement of DMARDs in the development and progression of this RA complication [40].

## Figures and Tables

**Table 1:** Demographic, clinical, and serological features of patients at baseline.

Demographic and Clinical Data	Result
Mean age	65 (11)
Female/Male ratio	2.7/1
Current/Ever smoker	6 (13.6%)/17 (38.6%)
COPD	8 (18.2%)
Disease duration	
ILD duration before ABA therapy (months)	20 (58)
Follow-up (months)	26.5 (38)
Rheumatoid factor	38 (86.4%)
ACPA	40 (90.1%)
HRCT pattern	
UIP	19 (43.2%)
NSIP	22 (50%)
CPFE	2 (4.5%)
OP	1 (2.3%)
Forced vital capacity (%)	88.5 (18.5)
Diffusion lung CO (%)	66.4 (34.5)
Use of cDMARDs before ABA	44 (100%)
Mehotrexate	32 (72.3%)
Leflunomide	20 (45.5%)
TNF*α* inhibitors	19 (43.2%)
Tocilizumab	9 (20.5%)
Rituximab	5 (11.4%)
Janus kinases inhibitors	3 (6.8%)
ABA monotherapy	11 (25%)
ABA + methotrexate	17 (38.6%)
Corticosteroids	33 (75%)
Continuous data are reported as median (IQR).	

COPD: chronic obstructive pulmonary disease; ILD: interstitial lung disease; ACPA: anti-cyclic citrullinated peptides antibodies; HRCT: high-resolution computer tomography; UIP: usual interstitial pneumonia; NSIP: nonspecific interstitial pneumonia; OP: organizing pneumonia; CPFE: combined pulmonary fibrosis and emphysema; cDMARDs: conventional disease-modifying anti-rheumatic drugs; ABA: abatacept; IQR: interquartile range.

**Table 2:** Demographic, clinical, and serological features of patients at baseline according to their HRCT outcome.

	HRCT Improved/Stable	HRCT Worsened	*p*
	36	8	
Mean age	64 (10)	67 (22)	0.94
Female/Male ratio	3/1	1.7/1	0.063
Smoking habit	7 (19.4%)/16 (44.4%)	1 (12.5%)/2 (25%)	0.36/0.63
COPD	8 (22.2%)	2 (25%)	0.55
Disease duration	120 (149)	87 (167)	0.87
ILD duration before ABA therapy (months)	14 (60)	26 (44)	26 (44)
Follow-up (months)	25 (29)	52 (49)	0.48
Rheumatoid factor	31 (86.1%)	7 (87.5%)	0.92
ACPA	40 (90.1%)	7 (87.5%)	0.57
HRCT pattern			
UIP	13 (36.1%)	6 (75%)	
NSIP	20 (55.5%)	2 (25%)	0.24
CPFE	2 (5.6%)	–	
OP	1 (2.8%)	–	
Forced vital capacity (%)	88 (17)	93 (22)	0.46
Diffusion lung CO (%)	61 (34)	81 (22)	0.14
Use of cDMARDs before TCZ	36 (100%)	8 (100%)	
Mehotrexate	26 (72.2%)	6 (75%)	0.87
Leflunomide	17 (47.2%)	3 (37.5%)	0.71
TNFi	16 (44.4%)	3 (37.5%)	0.72
Tocilizumab	8 (22.2%)	1 (12.5%)	0.54
Rituximab	3 (8.3%)	2 (25%)	0.22
Janus kinases inhibitors	2 (5.6%)	1 (12.5%)	0.46
ABA monotherapy	9 (25%)	2 (25%)	1
ABA + methotrexate	13 (36.1%)	4 (50%)	0.69
Corticosteroids	29 (80.6%)	4 (50%)	0.09

COPD: chronic obstructive pulmonary disease; ILD: interstitial lung disease; ACPA: anti-cyclic citrullinated peptides antibodies; HRCT: high-resolution computer tomography; UIP: usual interstitial pneumonia; NSIP: nonspecific interstitial pneumonia; OP: organizing pneumonia; CPFE: combined pulmonary fibrosis and emphysema; cDMARDs: conventional disease-modifying anti-rheumatic drugs; ABA: abatacept; IQR: interquartile range.
